# Determination of glyphosate in breast milk of lactating women in a rural area from Paraná state, Brazil

**DOI:** 10.1590/1414-431X2022e12194

**Published:** 2022-07-25

**Authors:** M. Camiccia, L.Z.P. Candiotto, S.C. Gaboardi, C. Panis, L.B.M. Kottiwitz

**Affiliations:** 1 Programa de Pós-Graduação em Ciências Aplicadas è Saúde, Universidade Estadual do Oeste do Paraná, Francisco Beltrão, PR, Brasil; 2 Laboratório de Biologia de Tumores, Universidade Estadual do Oeste do Paraná, Francisco Beltrão, PR, Brasil; 3 Grupo de Estudos Territoriais, Universidade Estadual do Oeste do Paraná, Francisco Beltrão, PR, Brasil; 4 Programa de Pós-Graduação em Geografia, Universidade Estadual do Oeste do Paraná, Francisco Beltrão, PR, Brasil; 5 Instituto Federal Catarinense, Campus Ibirama, Ibirama, SC, Brasil

**Keywords:** Herbicide, Agrochemicals, Human milk, Glyphosate, Environmental pollution

## Abstract

The aim of this study was to verify the presence of glyphosate in breast milk and to characterize maternal environmental exposure. Sixty-seven milk samples were collected from lactating women in the city of Francisco Beltrão, Paraná, living in urban (n=26) and rural (n=41) areas, at the peak of glyphosate application in corn and soy crops in the region (April and May 2018). To characterize the study population, socio-epidemiological data of the women were collected. To determine glyphosate levels, a commercial enzyme immunosorbent assay kit was used. Glyphosate was detected in all breast milk samples analyzed with a mean value of 1.45 µg/L. Despite some descriptive differences, there were no statistically significant differences (P<0.05) between the categories of the variables tested. Also, glyphosate was detected in drinking water samples from the urban area and in artesian well water from the rural area of the region where the studied population lived. The estimation of the total amount of glyphosate ingested by breastfeeding babies in a period of 6 months was significant. These results suggest that the studied lactating population was contaminated with glyphosate, possibly through continued environmental exposure.

## Introduction

The World Health Organization (WHO) recommends that all babies be fed exclusively on breast milk until sixth months of age. Thus, there is great concern about the contamination of this food (or substance) since children are more vulnerable due to the immaturity of their vital systems, including the immune system. Although breast milk has a high nutritional value, it can be an important source of chemicals for breastfeeding children, being a good indicator of environmental and maternal exposure due to its representative lipid fraction and the consequent presence of several xenobiotics (1).

The chemical contamination of human milk is widespread and results from decades of environmental contamination by toxic products. High levels of contaminants have been reported in women living in agricultural areas of developing countries with intensive pesticide use (2). Although the benefits of breastfeeding outweigh the risks of contaminants in human milk, the continuous identification of these compounds in the breast milk is extremely important so that public health measures can be taken to reduce this contamination (3).

Pesticides are considered important inputs for agricultural production, despite their effects on the environment, and human health has not been widely studied and disseminated to society (4). In recent years, Brazil has been the largest consumer of pesticides in the world. The public health impacts are widespread, reaching vast territories and affecting different populations, such as workers in different activities, residents of factories and farms, and all consumers of contaminated food and water. Among pesticides, glyphosate is the market leader in Brazil, accounting for 33.6% of the total pesticides sold (5).

In this context, the municipality of Francisco Beltrão is not far from the reality of the country. The city has a strong agricultural activity, especially soybeans and corn monocultures, which require treatment with herbicides, among which glyphosate and 2,4D are the most commonly used (6). Given the development model of Brazilian agriculture, which is based on the growing demand for synthetic chemical agents, studies that analyze the impact of pesticide use on the population are relevant to measure the development of those affected. Thus, this research aimed to investigate the presence of the herbicide glyphosate in the breast milk of lactating women living in the city of Francisco Beltrão, Paraná, during the peak of spraying of this substance in the region, to measure the resulting environmental contamination, and to assess the association between the socio-environmental parameters of this herbicide and the health history of lactating women.

## Material and Methods

### Study design and scenario

This was a cross-sectional study with data collected in a single moment from lactating women of the city of Francisco Beltrão in April and May 2018. During this period, the harvest of corn crops (transgenic, planted in January) and the desiccation of soybean residues occur, with spraying of the herbicide glyphosate.

**Figure 1:**
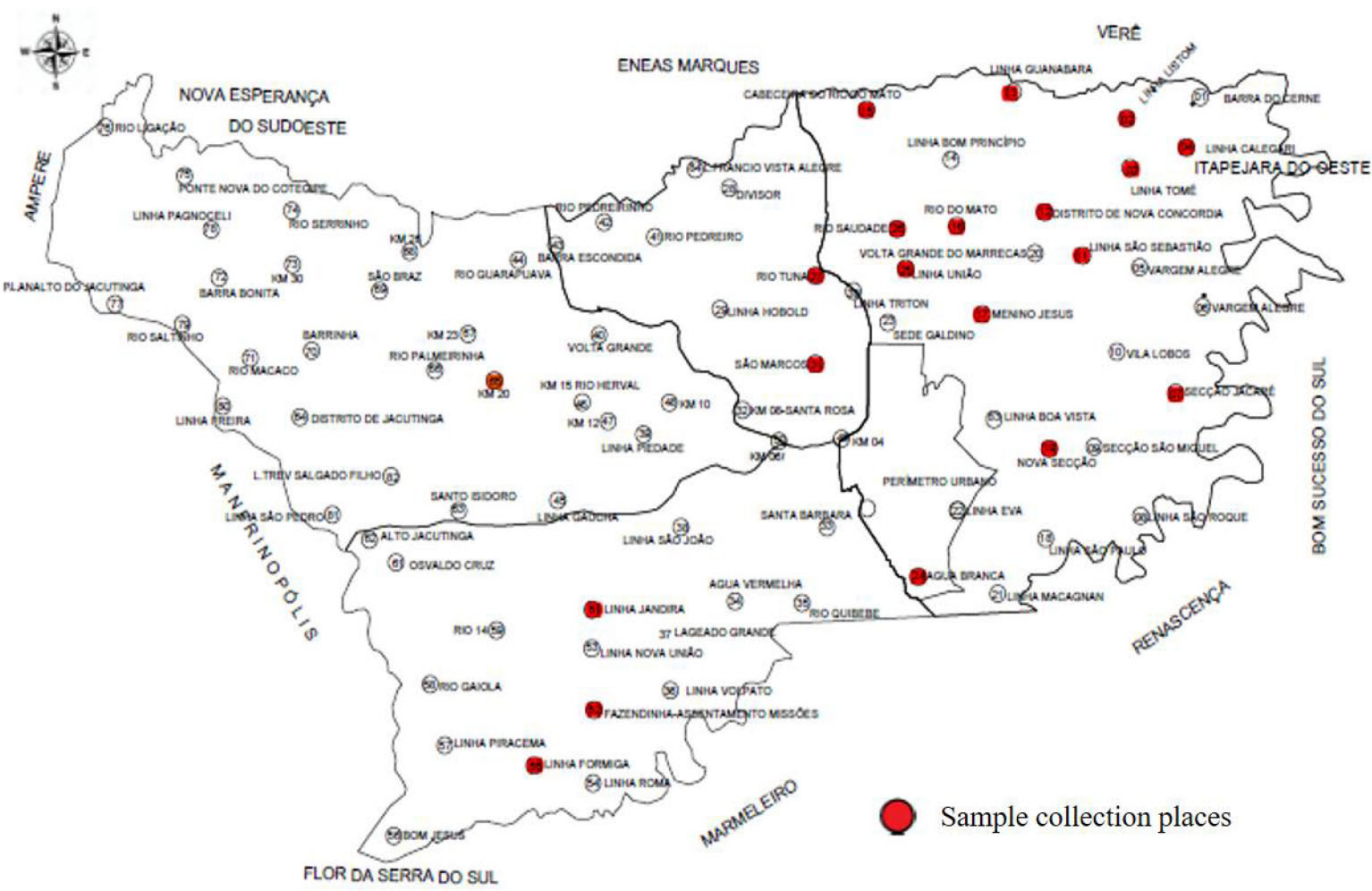
Localities selected for sample collection in the city of Francisco Beltrão, Paraná state, Brazil.

The inclusion of lactating women was made through the Family Health Strategy (FHS) program of the city (Figure 1). The present study included mothers who have lived in the municipality for at least one year and were in different phases of breastfeeding.

### Ethical aspects

The signing of a free and informed consent form (ICF) was requested, confirming the voluntary acceptance to participate in the study. The research was approved by the Human Research Ethics Committee (CEP) of UNIOESTE and under the consubstantiated opinion of the CEP: 2.588.616 of April 9, 2018, in compliance with resolution 466/12 of the Ministry of Health.

### Data and sample collection

Patients were interviewed to obtain general demographic information and data about the newborn's gestational period, husband/partner, mother's home and occupation, work environment, and pesticide exposure. We sought to characterize the participating mothers, know their life habits, and associate this information with the presence of pesticide residues in breast milk. Detailed information is shown in Table 1.

**Table 1:** Socio-demographic characteristics of lactating women enrolled in the study.

	Mean	SD
Age (years)	27.3	5.7
Weight (kg)	65.5	10.4
Height (m)	1.62	0.7
Body mass index (kg/m^2^)	24.7	3.8
Child age (months)	6.7	7.1
Number of pregnancies	2.0	1.1
Number of abortions	0.2	0.5
Number of prenatal appointments	12.0	3.4
Distance from house to crop (m)	166.4	251.2
Time living in rural area (years)	7.3	6.5
Glyphosate levels in milk (ppb)	1.45	0.1
	n	%
Type of residence		
Urban	26	38.8
Rural	41	61.2
Type of delivery		
Cesarean	41	61
Normal	26	39
Marital status		
Single	5	7.5
Legally married	39	58.2
Living together	23	34.3
Race		
White	52	77.6
Black	15	22.4
Education level		
Incomplete primary education	11	16.4
Complete primary education	18	26.9
Complete high school	22	32.8
Complete higher education	16	23.9
Occupational status		
Home worker	18	26.9
Farmer	12	17.9
Other	37	55.2
Works close to the crops		
Yes	35	52.2
No	32	47.8
Lives in the rural area		
Yes	49	73.1
No	18	26.9
Works in the rural area		
Yes	30	44.8
No	37	55.2
Works at the crops		
Yes	30	44.0
No	38	56.0
Works in pesticide spraying		
Yes	11	16.4
No	56	83.6
Has a food garden at home		
Yes	35	52.2
No	32	47.8
Sprays pesticide in the food garden		
Yes	35	52.2
No	32	47.8

SD: standard deviation; n: number of individuals.

A total of 67 samples of breast milk were collected from lactating women living in rural (n=41) and urban (n=26) areas, through manual compression of the breast in a single collection. In the rural area, the collections were carried out at the FHS of Nova Concórdia, Assentamento Missões, and KM 20. In the urban area, sample collection took place at the Health Center of the North City, where public pediatric care is carried out. A volume between 2 and 10 mL, from one of the volunteers’ breasts, was collected directly in sterile glass test tubes with a rubber stopper. Subsequently, the samples were identified and kept frozen at the Tumor Biology Laboratory, at the State University of Western Paraná, Francisco Beltrão campus, until analysis.

To calculate the sample size (7), we considered that the number of children born in Francisco Beltrão in 2017 was 1,309, approximately 41% of them were breastfed, and the collections were performed in two months in 2018 (February/March). The estimated sample for this research was 72 children.

### Glyphosate measurement in breast milk and water samples

The analytical determination of glyphosate levels in breast milk samples and in water samples from artesian wells in the rural area (n=6) was conducted by enzyme-linked immunosorbent assay (ELISA) as described by Nardo et al. (8) and adapted according to the manufacturer's instructions (Abraxis LLC, USA) for biological matrices. Intra- and inter-experiment analyses were performed on control samples to assess reproducibility and analytical variation. Samples were pre-concentrated on Millipore columns (Centrifugal filters, Millipore, USA) by centrifugation at 4400 *g* (25°C, 15 min). For the detection and quantification by ELISA, we used a glyphosate detection kit (Abraxis LLC).

Samples and analytical standards provided in the kit were derivatized and added to the microplate wells for incubation and analysis at 450 nm, using a microplate reader (Polaris, CELER Biotecnologia, Brazil). Sample concentrations were determined by interpolation with the standard curve. This method has a detection limit of 0.05 µg/L and a quantification limit of 0.013 µg/L, with a maximum detection concentration of 4 µg/L.

For urban area samples, glyphosate levels in drinking water were obtained from a report provided by the 8th Regional Health Department of Paraná, which belongs to the municipality of Francisco Beltrão, for the same period of milk sample collection. The water samples collected along the Marrecas river basin (which supplies this region) at 12 different points were evaluated by an outsourced laboratory using chromatography coupled to mass spectrometry. Samples from the rural area were collected from artesian wells of properties along the Marrecas river basin. The same method used to measure milk samples (ELISA) was applied here.

### Statistical analysis

The data were tabulated and descriptive statistics were used to determine the mean, standard deviation, and frequency distribution of the data obtained by the questionnaires. For the association between the questionnaire data and the results of the ELISA tests, the chi-squared test was used with a significance level of 5% (P<0.05), using Microsoft Windows 10 and the SPSS program version 24 (IBM, USA), using glyphosate levels in breast milk as a dependent variable.

## Results

The general characteristics of the sample are shown in Table 1. The participants were young adults and lived in the current residence for an average of seven years. Only 25% of them had completed higher education. Most lived in the countryside but did not work in the fields. Less than half of the participants worked in the rural area, 44.7% (n=30), of which just over 16% used pesticides. Approximately 18% were farmers. As for the parents of the infants, 67% (n=45) had activities in the city.

Considering the habits and health of the breastfeeding women, 93% reported that they did not smoke, 39% did not use any type of medication, 6% had been victims of pesticide poisoning, 52% used household pesticides, 72% lived close to crops, and 60% had home gardens (Table 2). Considering only infants aged 0 to 6 months, 78% of the mothers reported exclusive breastfeeding and 22% did not.

**Table 2:** Glyphosate levels in breast milk samples from lactating women enrolled in the study according to socio-demographic variables.

	Mean (ppb)	SD	P value
Type of residence			
Urban	1.47	0.14	0.219
Rural	1.43	0.07	
Race			
White	1.45	0.10	0.956
Black	1.45	0.12	
Education level			
Incomplete primary education	1.43	0.01	0.186
Complete primary education	1.49	0.17	
Complete high school	1.42	0.01	
Complete higher education	1.47	0.11	
Occupational status			
Home worker	1.44	0.10	0.582
Farmer	1.43	0.02	
Other	1.46	0.12	
Works close to the crops			
Yes	1.45	0.10	0.842
No	1.45	0.11	
Lives in the rural area			
Yes	1.44	0.06	0.082
No	1.50	0.17	
Works in the rural area			
Yes	1.45	0.11	0.759
No	1.44	0.10	
Works at the crops			
Yes	1.44	0.08	0.395
No	1.46	0.12	
Works in pesticide spraying			
Yes	1.47	0.13	0.557
No	1.45	0.10	
Has a food garden at home			
Yes	1.45	0.09	0.770
No	1.46	0.12	
Sprays pesticide in the food garden			
Yes	1.46	0.12	0.722
No	1.45	0.08	

SD: standard deviation; ppb: parts per billion (µg/L). Chi-squared test (P>0.05).

**Figure 2:**
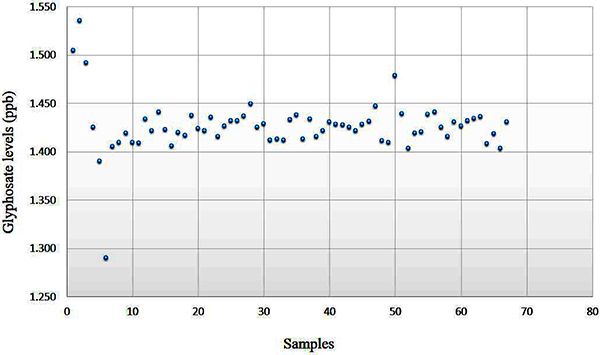
Glyphosate levels detected in 67 breast milk samples from the lactating women enrolled in the study (ppb in µg/L).

We detected glyphosate residues in all 67 breast milk samples analyzed (Figure 2). The average level was 1.45 µg/L (Table 2). There was little variation in glyphosate level between different categories (1.42 to 1.50 µg/L), and there were no statistically significant differences (P>0.05) between the variables tested. Table 1 demonstrates that it was not possible to establish an association between glyphosate in breast milk samples and place of residence of the nursing mothers or even working (past or present) in the fields/countryside (P>0.05). Table 3 shows the estimated volume of glyphosate ingestion by the babies according to their age/weight and daily breast milk intake. The highest amount of glyphosate ingested by a child at 6 months was 255.6 micrograms. Water analysis (artesian well and drinking water) showed glyphosate at average levels below 0.001 and 0.802 µg/L, respectively.

**Table 3:** Estimated volume of glyphosate intake from breast milk by infants according to age in months, average weight, and volume of breast milk ingested in each age group.

Age, weight	Total volume of breast milk ingested (L)	Estimated quantity of glyphosate ingested (µg)
1 month, 4 kg	18.30	26.53
2 months, 5.5 kg	25.17	36.50
3 months, 6.5 kg	29.80	43.21
4 months, 7 kg	32.04	46.45
5 months, 7.5 kg	34.33	49.77
6 months, 8 kg	36.62	53.10
Total	175.26	255.56

## Discussion

The study of the relationship between exposure to pesticides and their presence in biological fluids such as breast milk is of great interest to public health. Our study aimed to evaluate the levels of glyphosate, the most widely used herbicide in southwestern Paraná, Brazil, in the breast milk of breastfeeding women at the peak of its pulverization period and characterize the environmental contamination by this pesticide. Our findings showed contamination by glyphosate in all analyzed breast milk samples and in water samples collected from the same region of the studied breastfeeding women.

Regarding the profile of the lactating women, most were exclusively breastfeeding (EBF). This indicated that the primary health care service was being carried out effectively in this community, promoting this practice as one of the priorities in public health actions. Further, it showed that these breastfeeding women were aware of the importance of EBF, which is fundamental for the child's health. It also reinforces an important advance in the Brazilian health system, especially compared to developed countries where the EBF adherence rate in children up to 6 months is below 16% (9). These findings highlight that the current policies to encourage EBF are effective compared to other municipalities in the country with a similar population (10).

Undoubtedly, the most relevant finding of our study was the detection of glyphosate in all breast milk samples evaluated. The distribution of levels in µg/L was quite similar among the sub-samples. There is no legislation about the minimum levels of glyphosate in human milk, but in the case of a pesticide, we must assume that the acceptable level is zero. Thus, it becomes difficult to estimate the impact of glyphosate consumption on the infant.

We calculated the putative cumulative glyphosate intake of these children based on the values identified in breast milk. A recently published literature review (11) proposed an equation to estimate a baby’s breastfeed intake per day and provided a volume of 152.6 mL·kg^-1^·day^-1^. Thus, following age-adjusted WHO weight data in childhood, the values shown in Table 3 describe 30-day consumption of breast milk. The data showed that an infant breastfed for six months would ingest an estimated total dose of 256 µg/L of glyphosate. This dose, even cumulatively, does not represent a toxic accumulation of glyphosate since it must be greater than 1.75 mL·kg^-1^·day^-1^ to cause damage (12).

Mothers' contact with pesticides through their contaminated husbands or partners can cause milk contamination (13). It is important to say that other sources of contamination can explain the similar levels of glyphosate contamination in milk samples from lactating women living in rural and urban areas observed here. Less than 0.3% of pesticides applied reach their target. In this way, a large part of the sprayed pesticide can be dispersed in the different environmental compartments: air, soil, and water (14).

Studies have sought to understand the consequences of glyphosate exposure during breastfeeding. Experimental data show that male rats fed glyphosate-complemented soymilk present endocrine disruption characterized by reduced testosterone levels, impaired number of Sertoli cells, and decreased spermatids (15). Also, Dallegrave et al. (16) demonstrated that lactational exposure to glyphosate in animals impacts their reproductive system in puberty and adulthood by causing a reduction in sperm counts and testosterone levels. Such studies corroborate others that discuss glyphosate as an endocrine-disrupting chemical (17-19). These findings indicate the potential of glyphosate for endocrine disruption and reinforce the need for more studies to understand the implications of its chronic exposure through lactation on child development.

Our findings on environmental contamination showed that the observed levels of glyphosate in water are within the levels allowed by the Brazilian law for this substance (up to 500 µg/L). However, when we look at the maximum limits authorized by countries in the European Union (0.1 µg/L per pesticide) (20), these findings are of concern, especially considering the levels found in artesian well water. This finding reinforces the occurrence of glyphosate-contaminated breast milk samples in a population living in a geographic region with glyphosate in the water. Various pesticides enter water resources and contaminate humans (21). At the national level, Consolidation Ordinance No. 05 of September 28, 2017 of the Ministry of Health establishes a maximum limit of 500 µg/L for the sum of glyphosate and AMPA compounds in water intended for human consumption (22).

The main routes of dispersal for glyphosate in water are microbiological degradation and association with sediments. Glyphosate does not degrade quickly in water, but in the presence of aquatic microflora, glyphosate decomposes into AMPA and eventually into carbon dioxide. AMPA toxicity is equal to or greater than glyphosate itself (23). Since glyphosate-AMPA is mobile in the environment, its presence in surface and groundwater is likely to increase animal and human exposure (24). The presence of glyphosate in food, although in low concentrations, suggests that glyphosate persists in the food chain "beyond the farm gate" throughout the commercial market, at all stages of storage, transportation and processing, preparation, and finally consumption (25).

In the present study, glyphosate was detected in human breast milk at the peak of pulverization season. Also, our findings indicated glyphosate residues in water samples from the same region of breastfeeding women, suggesting that environmental contamination could contribute in part to the pesticide load in human milk. Considering that the impact of pesticides on health has been documented in Brazilian studies from the same geographical area (26-28) and global efforts to support infant breastfeeding (29,30), these findings contribute significantly to this issue. Monitoring actions are necessary for this population since the consequences of glyphosate in child development are unclear.
